# Supplementary figures and images for: Extracellular vesicles secreted by Giardia duodenalis regulate host cell innate immunity via TLR2 and NLRP3 inflammasome signaling pathways
Source: PLoS Negl Trop Dis. 2021 Apr 2;15(4):e0009304. doi: 10.1371/journal.pntd.0009304 (PMC8046354; doi:10.1371/journal.pntd.0009304)

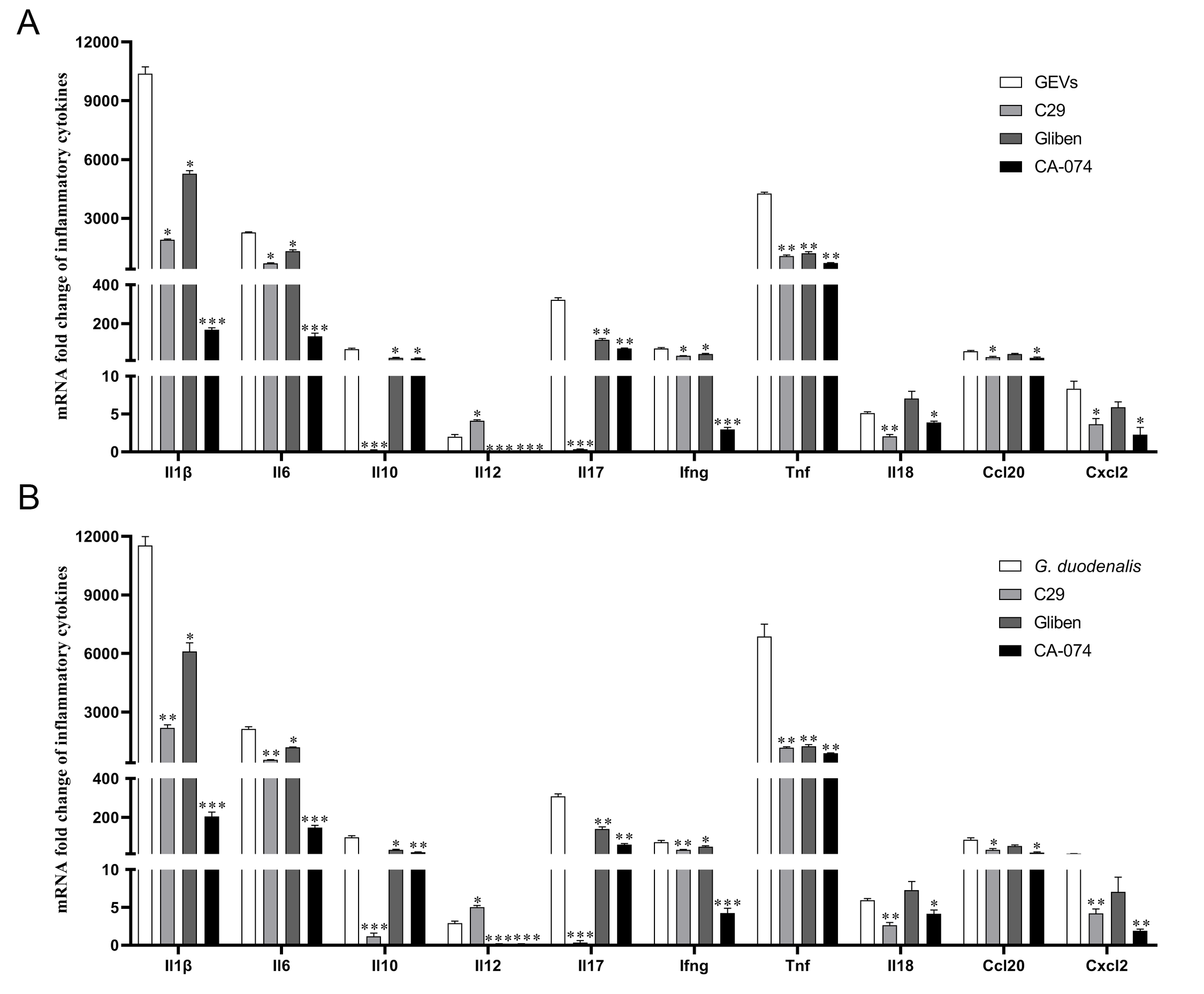

Supplement: S1 Fig — Murine peritoneal macrophages were pre-treated with 100 μM C29, 100 μM Glibenclimide, or 25 μM CA-074 Me for 1 h and then added 25 μg/mL GEVs or 1.5 × 10^6^ parasites/mL G. duodenalis for 12 h. PBS-treated was used as negative control. Cells were collected and the mRNA expression levels of cytokines were measured using qPCR assays. (TIF) [file pntd.0009304.s001.tif]
