# Supplementary material for: Extracellular vesicles secreted by Giardia duodenalis regulate host cell innate immunity via TLR2 and NLRP3 inflammasome signaling pathways
Source: PLoS Negl Trop Dis. 2021 Apr 2;15(4):e0009304. doi: 10.1371/journal.pntd.0009304 (PMC8046354; doi:10.1371/journal.pntd.0009304)
Supplement: Attachment — Submitted filename: Giardia PNTD.docx [file pntd.0009304.s005.docx]

The manuscript Extracellular vesicles secreted by Giardia duodenalis regulate host cell innate immunity via TLR2 and NLRP3 inflammasome signaling pathways by Zhao et al. is an interesting work that must be improved to be accepted in PNTD.

The authors in their introduction and formulation of hypotheses suggest that there are no works describing extracellular vesicles and aspects of innate immunity in Giardia intestinalis. They did not consider the contribution of 3 groups from Swedish, Brazilian and Argentine citing exosomes and microvesicules of G intestinalis and its intearaction with the host. This information has been considered in at least 5 reviews in the parasite-host cell field and the first EV report in G intestinalis was four years ago!!!Even more the production and purification of EVs in the manuscript is unusual and nuclear.

I also recomend that the group read a paper position of the international socitey of extracellular vesicles with the minimum requirements to characterize [EVs.The](https://ssl.microsofttranslator.com/bv.aspx?ref=TAns&from=&to=en&a=EVs.The) authors show Nano tracking data however they did not perform the reading of 3 measurements for one minute which is the normal procedure. The data presented is not convincing being difficult to accept.

Uptake trials in macrophages are not convincing too. In general Extracelular vesicles are made up of highly fuseable molecules and the internalizacao processes are very fast. Moreover Evans Osses et al 2017, Gavinho et al, 2020 have described uptake to eukariotic cell with reduzed amount of Gevs .25 ug de Evs is a huge concentration of EVs. Other authors have already shown na intense internalization with less GEVs

In general the authors must justify because they used 25 ug of vesicles. In tempo 0 and at an hour of incubation there should be Evs at the plasmatic membrane. The controls at the internalization should not be PBS and should be endocitocis inhibitors like cythochalasin to demonstrate that the vesicles enter through passive or active processes. 25 ug is a lot of EVs and the data may be indicating that there are problems in the PKH labeled of EVs as already indicated by gavinho et al, 2020

The authors should justify because they work with 25 ml ug in all experiments and try to do experiments with different doses in order to show dose dependence effect .

Again the authors did not cite the literature when they show pro-inflammatory effects of EVS release. It is known that giardiasis takes place with low cytokine release and not pro inflamatory release . There are still manuscript on the effect of the secretoma of G.I against eukaryotic cell

The effect of GEVS And inflamasome should be done with more controls and with diferent concentrations of GEVS
