# Supplementary material for: Extracellular vesicles secreted by Giardia duodenalis regulate host cell innate immunity via TLR2 and NLRP3 inflammasome signaling pathways
Source: PLoS Negl Trop Dis. 2021 Apr 2;15(4):e0009304. doi: 10.1371/journal.pntd.0009304 (PMC8046354; doi:10.1371/journal.pntd.0009304)
Supplement: Attachment — Submitted filename: Response to reviewers-20210122.docx [file pntd.0009304.s006.docx]

**Methods**

Reviewer 1
Q1: my concern for authors not to be guided or cite important literature in the area. Experiments with macrophages and internalization must be improverd. Authors do not cite and do not know essential works in the area.

A1: Thank you for your precious comments. Several important literatures on *G. intestinalis* EVs or innate immune responses have been cited and in the introduction, results and discussion sections (highlighted in red). These supplemented publications are listed as follows:

Ref 26: Moyano S, Musso J, Feliziani C, Zamponi N, Frontera LS, Ropolo AS, et al. Exosome biogenesis in the protozoa parasite *Giardia lamblia*: a model of reduced interorganellar crosstalk. Cells. 2019;8(12): 1600.

Ref 30: Gavinho B, Sabatke B, Feijoli V, Rossi IV, Silva JM, Evans-Osses I, et al. Peptidylarginine deiminase inhibition abolishes the production of large extracellular vesicles from *Giardia intestinalis*, affecting host-pathogen interactions by hindering adhesion to host cells. Front Cell Infect Microbiol. 2020;10: 417.

Ref 31: Evans-Osses I, Mojoli A, Monguió-Tortajada M, Marcilla A, Aran V, Amorim M, et al. Microvesicles released from Giardia intestinalis disturb host-pathogen response *in vitro*. Eur J Cell Biol. 2017;96(2): 131-42.

Ref 36: Ma'ayeh SY, Liu J, Peirasmaki D, Hörnaeus K, Bergström Lind S, Grabherr M, et al. Characterization of the *Giardia intestinalis* secretome during interaction with human intestinal epithelial cells: the impact on host cells. PLoS Negl Trop Dis. 2017;11(12): e0006120.

We read two papers on the internalization experiments. Evans-Osses et al. reported that microvesicles released from *Giardia intestinalis* are captured by immature dendritic cells (iDCs) through assays of flow cytometry and confocal imaging. The plasma membrane vesicles (MVs) were labelled with PKH-67 and then added to iDCs. The percentage of PKH-67^+^ iDCs was analysed. This phenomenon was blocked by Cytochalasin-D. In addition, the MVs could be co-localized with proteins, such as early endocytosis associated protein. Ma’ayeh et al. reported that *Giardia intestinalis* excreted-secreted products (ESPs) were internalized to intestinal epithelial cells (IECs) by observing Alexa Fluor488-labelled ESPs location using confocal imaging assay. In our last manuscript version, we only pictured PKH-67 labeled GEVs in primary mouse peritoneal macrophages through confocal imaging assays at 1 h, 3 h, and 6 h. Considering those previous reports, we improved our *Giardia duodenalis* extracellular vesicles (GEVs) internalization assays as follows. First, we inoculated 25 μg of PKH67 labeled GEVs into macrophages and incubated for 1 h, 3 h, and 6 h and quantified the PKH67 positive cells percentage using flow cytometry. We found that PKH67-labeled GEVs could entered into macrophages in a time-dependent manner. Moreover, most GEVs were rapidly internalized into hosts within 3 h (79.2%). Then, we compared different doses of GEVs (25 μg or 12.5 μg) internalization into host cells and found that GEVs entered into macrophages in a dose-dependent manner. Finally, we determined the way of GEVs entrance into macrophages, cells were pretreated with Cytochalasin-D, then add labeled GEVs and observed with laser confocal. We found that GEVs may probably be captured by macrophages through active phagocytosis.

Reviewer 2
Q1: Line 330: LAL assay measures LPS which is not present in Giardia EVs. Also, the relevance of LAL values should be mentioned in the discussion not in the results section. LAL is not the right method here as it measures LPS in gram negative bacteria.

A1: Thank you for your precious comments. We have deleted LAL assay in the method and result sections.

Q2: Lines 331: 25 ug/mL Did the authors do a BCA to quantify protein content? How did the authors isolate 25 ug/mL? Is it a physiological concentration? A range of concentrations should be used.

A2: Thank you. The protein contents in the GEVs were quantified with a BCA Protein Assay Kit (Thermo Scientific, USA). This has been specified in the method section (line 150-151). The concentrated GEVs after ultracentrifugation was about 3.7 µg/µL. We also supplemented other dose of GEVs-treated experiments and compared the roles of different doses of GEVs (25 μg or 12.5 μg) in triggering inflammatory response. Results are added in line 363 and figure 3.

Q3: Line 336: Did the authors use a positive control? How about Giardia trophozoites?

A3: Thank you. Previous study by Li et al. showed that Giardia trophozoites could enhance proinflammatory cytokines of IL-6 and TNF-α production in wild type mouse peritoneal macrophages; thus, Giardia trophozoites could be used as a positive control. We have added Giardia trophozoites-treated group as positive control in this assay and results are added in line 365 and figure 3.

Ref: Li X, Zhang XC, Gong PT, Xia FF, Li L, Yang ZT, et al. Tlr2−/− mice display decreased severity of giardiasis via enhanced proinflammatory cytokines production dependent on AKT signal pathway. Front. Immunol. 2017;8: 1186.

Q4: In the methods section: The proteomic content of EVs was not analyzed. The authors should further characterize EVs protein content. (See Thery et al. article in JEV for guidelines about EVs research).

A4: Thank you. Proteomic of GEVs were added in the method section (line 163-173) and result section (line 311-325). Information of the identified 153 proteins were showed in Table S1; gene ontology annotations were showed in Table S2 and Figure 1C; KEGG analysis were showed in Table S3 and Figure 1D.

Q5: Figure 4, 5, 6, 7, 8, 9: Do the data illustrate “fold change” or “ratios”? No SEM is indicated in non treated groups. How did the authors do statistical analysis with Ratios?

A5: Thank you. Results of qPCR assays were fold change and the relative mRNA fold change was calculated as 2^-ΔΔCt^, where ΔCt represents the Ct (target gene) - Ct (actin) and ΔΔCt represents the ΔCt (sample) - ΔCt (control) (line 233-235). In the control group, the “ ΔCt (sample) - ΔCt (control)” was 0 and 2^-ΔΔCt^ was 1. So, there is no SEM in the control group. Thus, we deleted these control groups. Results of WB gray value were ratios and the protein gray value ratios were calculated as gray value (sample)/gray value (actin). The statistical analysis was carried out using one-way ANOVA.

Q6：Lines 31-32. Please specify the target selectivity and effects of the inhibitors.

A6: Thank you. CA-074 Me is an inhibitor of cathepsin B. zVAD-fmk is an inhibitor of pan-caspase. These information have been specified in line 30-31.

Q7：Line 38: anti-giardiasis should be reworded as anti-Giardia

A7: Thank you. Corrections have been made in line 39.

Q8：Line 50-52. Please rephrase these lines. This is not the first study to demonstrate the internalization of Giardia EVs. Refer to Evans Osses et al. 2017.

A8: Thank you. Corrections have been made as follows: “This study demonstrated that *G. duodenalis* EVs could be rapidly internalized into primary mouse peritoneal macrophages, regulate host cell innate immunity via TLR2 and NLRP3 inflammasome signaling pathways, and provide new targets against giardiasis” (line 49-52).

Q9：Line 86-88: Please rephrase. There is canonical and non-canonical NLRP3 activation.

A9: Thank you. Corrections have been made as follows: “NLRP3 inflammasome is most well-investigated for it could be widely activated by various particles, uric acid crystals, toxins, bacterials, viruses, as well as parasites through caspase-1 dependent canonical pathway or caspase-11 dependent non-canonical pathway” (line 89-90).

Q10：Line 90. A reference is needed for the statement regarding the first signal in the stimulation of NLRP3.

A10: Thank you. Reference of a review on the mechanism of NLRP3 inflammasome activation has been added (line 92).

Q11：Lines 100-102: Please rephrase.

A11: Thank you. This sentence has been deleted and rephrase in line 103-117.

Q12：Please remove “of” before the names of the parasites. Same for Line 105.

A12: Thank you. Corrections have been made as suggested (line 107 and 109).

Q13：Line 109-113: Please rephrase. Also introduce the term GEVs before abbreviating it.

A13: Thank you. *G. duodenalis* EVs (GEVs) has been mentioned before abbreviating. This paragraph have been rephrased as follows: In the present study, we characterized *G. duodenalis* EVs (GEVs), verified GEVs could regulate immune response by triggering inflammatory cytokines transcription and secretion in hosts, investigated the roles of TLR2 and NLRP3 inflammasome signaling pathway in GEVs induced inflammatory response in murine macrophages and revealed the underlying mechanisms in *G. duodenalis*-host interactions (line 118-122).

Q14：Line 111: “Invested” should be replaced with “investigated”.

A14: Thank you. Corrections have been made as suggested (line 120).

Q15：Line 127 : How is an anaerobic environment created? Also, the term bottles is confusing. Were tubes used? Please note that Giardia is microaerophilic.

A15: Thank you. Sorry for our unclear description. The “12.5 mL bottles” has been replaced with “under microaerophilic conditions” (line 137). The *G. duodenalis* were cultured in polystyrene tubes and placed flatwise with 15 degree angle.

Q16：Line 132: The protocol cited for the isolation of EVs does not refer to Giardia EVs isolation.

A16: Thank you. References on *Giardia* EVs isolation (ref-26 and ref-31) have been added in line 143.

References:

Ref 26: Moyano S, Musso J, Feliziani C, Zamponi N, Frontera LS, Ropolo AS, et al. Exosome biogenesis in the protozoa parasite *Giardia lamblia*: a model of reduced interorganellar crosstalk. Cells. 2019;8(12): 1600.

Ref 31: Evans-Osses I, Mojoli A, Monguió-Tortajada M, Marcilla A, Aran V, Amorim M, et al. Microvesicles released from Giardia intestinalis disturb host-pathogen response *in vitro*. Eur J Cell Biol. 2017;96(2): 131-42.

Q17：Line 134: Add a reference of the product. (Exosome depleted FBS).

A17: Thank you. We added other papers that used the product of exosome-depleted fetal bovine serum (line 145, ref-33).

Q18：Line 150: The term tachyzoites (Toxoplasma) is used. Instead, it should be trophozoites.

A18: Thank you. We have corrected tachyzoites to trophozoites throughout this paper.

Q19：Line 157: Can you please describe the NTA protocol in detail like what camera settings, threshold etc. were used.

A19: Thank you. The detailed NTA protocols were as follows: The particle size and number of GEVs were measured using Nanoparticle tracking analysis (NTA). In detail, fresh GEVs were diluted to 1 × 10^7^ particles/mL in PBS, subjected to a ZetaView PMX 110 instrument (Particle Metrix, Germany), imaged 30 frames per second and lasted for 1 min. Samples were measured in triplicate at 25°C. Data was analyzed using ZetaView 8.02.28 software. (line 158-162). Parameters of camera shutter , gain or threshold were not constant for each sample, so we do not list these data.

Q20：Line 165-169: The protocol for labelling EVs using PKH-67 is based on Evans-Osses et al. and it should be cited.

A20: Thank you. Corresponding reference has been added (line 183).

Q21：Line 188: Please indicate the time point for stimulation of macrophages.

A21: Thank you. The stimulation time has been added (line 200).

Q22：Line 193: Please describe the protocol for macrophages/Giardia Transwell. Which media was used? Note that Giardia can stress the cells.

A22: Thank you. Yes, Giardia can stress the cells. To exclude this influence and explore the effects of GEVs, we used noncontact culture system to establish the positive control with *G. duodenalis*. The detailed protocol was as follows: “The transwell insert (Thermo Scientific, USA) with a membrane pore size of 0.4 micron was placed in cell plates coated with murine peritoneal macrophages, and *G. duodenalis* were added into transwell inserts culturing in RPMI 1640 medium with 2% fetal bovine serum” (line 202-205).

Q23：Line 217 (Table 1) Please follow the nomenclature for murine genes (ex: Il10 instead of IL10).

A23: Thank you. We checked the nomenclature for murine genes in NCBI and corrected in Table 1 (line 226).

Q24：Lines 234: Please change the title. (Inflammasome molecules should be removed.)

A24: Thank you. Corrections have been made as suggested (line 243).

Q25：Lines 276-279 (Statistical analysis section) : How was the normality of the data assessed? What kind of post-tests have been used? No T-test is used? Was the data parametric or non-parametric.

A25: Thank you. All the data in this study fit normal distribution and are parametric. Differences between two groups were analyzed by t-test and multiple groups were used analyzed by one-way ANOVA. The homogeneity of variance of data are analyzed by Levene test. Data of homogeneity of variance were further analyzed using post-test of bonferroni(B) and heterogeneity of variance were further analyzed using Tamhane’s T2(M). This has been described in detail in line 294-301.

Q26：Line 282: Please rephrase the title of the section. This has been shown and characterized in previous studies.

A26: Thank you. The title has been corrected to “Characterization and proteomic analysis of GEVs” (line 304).

Q27：Line 289: Can you indicate the diameter (mean) on Figure 1D.

A27: Thank you. We made three experiments to measure the mean diameter of GEVs was 144.8±0.99 nm and figure 1D was one of the results. To make accurate description, we replaced “144.8±0.99 nm” with “mean diameter at X50 was 143.5 nm” and replaced “4.63±0.07 × 10^10^ particles/mL” with “4.7 × 10^10^ particles/mL” (line 310). The attached is the original data.

Q28：Line 290: Figure 1C should be removed. NTA images are not relevant.

A28: Thank you. We have deleted figure 1C.

Q29：Line 390: Is Glibenclimide NLRP3-selective?

A29: Thank you. Glibenclimide is an inhibitor of NLRP3 by inhibiting K^+^ efflux. This has been sepcified in line 487.

Q30：Line 391: CA-074-Me is not a direct inhibitor of NLRP3.

A30: Thank you. We corrected “NLRP3 inhibitor of CA-074-Me” into “cathepsin B inhibitor of CA-074 Me” (line 490). The NLRP3 inflammasome could be activated by cathepsin B pathway and inhibited cathepsin B could then inhibit NLRP3.

Q31：Line 428: Why did the authors not use different concentration of EVs before? i.e. in figures 4,5 and 6.

A31: Thank you for your suggestion. We supplemented these assays and added different concentrations of EVs, including 25 μg/mL, 12.5 μg/mL in the revised figure 2, 3, 4, 5.

Q32：Line 431: The word “platform” does not fit here.

A32: Thank you. The word “platform”has been deleted as suggested (line 447).

Q33：General comment: The authors use ratio instead of doing to fold change and compare it to control. Also, no SD/SEM bars on the controlPlease edit.

A33: Thank you. Many papers showed the qPCR results as mRNA fold change/relative RNA levels without control displayed (ref-61, ref-36), whereas the WB gray value results as ratios. Results of qPCR assays were fold change and the relative mRNA fold change was calculated as 2^-ΔΔCt^, where ΔCt represents the Ct (target gene) - Ct (actin) and ΔΔCt represents the ΔCt (sample) - ΔCt (control). In the control group, the “ ΔCt (sample) - ΔCt (control)” was 0 and 2^-ΔΔCt^ was 1. So, there is no SD/SEM bars in the control group. Thus, we deleted the control bars. Results of WB gray value were ratios and the protein gray value ratios were calculated as gray value (sample)/gray value (actin).

Ref 36: Ma'ayeh SY, Liu J, Peirasmaki D, Hörnaeus K, Bergström Lind S, Grabherr M, et al. Characterization of the *Giardia intestinalis* secretome during interaction with human intestinal epithelial cells: the impact on host cells. PLoS Negl Trop Dis. 2017;11(12): e0006120.

Ref 61: Wang XC, Gong PT, Zhang X, Wang JL, Tai LX, Wang X, et al. NLRP3 inflammasome activation in murine macrophages caused by *Neospora caninum* infection. Parasit Vectors. 2017;10(1): 266.

Q34：Line 477: zVAD-fmk: Please introduce this term in this section. It inhibits caspase at the first place.

A34: Thank you. Corrections have been made as suggested (line 526).

Q35：Lines 484-486: Increase of IL-1 beta is not observed in vivo in mice infected with G. duodenalis.

A35: Thank you. We added “*in vitro*” in this sentence (line 535).

Q36：Lines 508-510: Rephrase.

A36: Thank you. This sentence has been rephrased as follows: “The present study characterized GEVs from *G. duodenalis* and explored the effects of GEVs on host cell innate immunity using primary mouse peritoneal macrophages and its potential mechanisms” (line 556-558).

Q37：Lines 525-526: The cytokines increased are NF-kB cytokines.

A37: Thank you. This sentence has been rephrased as follows: “Results showed that many NF-κB-mediated cytokines or chemokines, including Il6, Tnf, and Il1β, Il18, Il12, Ifng, Ccl20 and Cxcl2, involved in the process” (line 589-591).

Q38：Lines 527: We can’t really conclude about Th1/Th2 balance as the cytokines are observed in macrophages. Have the authors used NF-kB inhibitors?

A38: Thank you for your suggestion. We deleted these discussion (line 591). We do not use NF-κB inhibitors here. But we used NF-κB inhibitors in studying the GEVs induced-other signaling pathways activation. Here, we mainly want to show the roles of NLRP3 inflammasome and TLR2. so we only used inhibitors of NLRP3 inflammasome and TLR2.

Q39：Line 531: What is the role of IL-10? Pro-resolving

A39: Thank you. IL-10 is an anti-inflammatory cytokine (line 592-595).

Q40：Lines 528-533: How about the apoptosis and necropsy assays?

A40: Thank you. Previous studies by Certad et al. showed that *Giardia* can trigger enterocyte apoptosis, activated roapoptotic caspase-3 and -9, increased expression of proapop-totic Bax, decreased expression of antiapoptotic Bcl-2, and induced proteolytic cleavage of poly (-ADP-ribose) polymerase (PARP), finally induced alterations in the enterocyte tight junctions by breakdown/relocalization of proteins associated with these structures. Causing these genes or proteins have not been determined in our assays, so we do not discussion the relation with apoptosis and necropsy. But NLRP3 inflammasome may probably trigger cell death of pyroptosis with active caspase 1 p20, moreover, KEGG analysis of GEVs showed that some proteins in GEVs may lead to necroptosis, thus, our group explored the expression of pyroptosis execution protein of GSDMD and found that GEVs indeed mediated pyroptosis in macrophages. This would be reported in detail after research in depth.

Ref: Certad G, Viscogliosi E, Chabe M, Caccio SM. Pathogenic mechanisms of *Cryptosporidium* and *Giardia*. Trends in Parasitology. 2017;33: 561-76.

Q41：Line 550: “Immune inflammatory response” could be reworded.

A41: Thank you. “Immune inflammatory response” has been reworded into “Immune response” (line 617).

Q42：Lines 553-561: Please adjust the wording and rephrase.

A42: Thank you. These sentences have been rephrased.

Q43：Line 588: Authors also need to cite Manko et al. 2020 (International journal of parasitology)

A43: Thank you. Corrections have been made as suggested (line 653).

Q44: Lines 590. Glyburide NLRP3 inhibitor is commonly used to inhibit NLRP3 inflammasome activation. Why did Glyburide not used in this study?

A44: Thank you. Glibenclamide is also named Glyburide, they share the same CAS No. 10238-21-8.

Q45: Line 603 “environmental hazardous” should be reworded.

A45: Thank you. Corrections have been made as suggested (line 669).

Q46: Line 605-607: Activation of NLRP3 was first demonstrated by Manko et al IJP 2020. The novel findings is the role of EVs.

A46: Thank you. Corrections have been made in line 669-674.

Reviewer 3

Q1: Line 127. Is it bottles or tubes? Was it anaerobic or microaerophilic?

A1: Thank you. Sorry for our unclear description. The “12.5 mL bottles” has been replaced with “under microaerophilic conditions” (line 137). The *G. duodenalis* were cultured in tubes (line 139).

Q2: Line 133. What volumes/number of cells were used in the preparations of EVs?

A2: Thank you. The *G. duodenalis* trophozoites were diluted into 1 × 10^6^ parasites/mL in modified TYI-S-33 medium with 12.5% exosome-depleted fetal bovine serum (line 143-144).

Q3: Giardia has no tachyzoite stage, it is trophozoites! This is elementary knowledge and the same mistake is repeated through-out the paper.

A3: Thank you. We have corrected tachyzoites to trophozoites throughout this paper.

Q4: Line 160. How pure is this cell population? Are there other immune cells like mast cells, DCs, neutrophils

A4: Thank you. The purity of cells were 99.1%. The cell purity was verified using flow cytometry through staining with APC anti-mouse/human CD11b (1:200, BioLegend, USA). The attached was the result of cell purity. This has been specified in the method section (line 180-181) and discussion section (line 587). The attached was the original data.

**Results**

Reviewer 1

Q1: The Ev characterizathion and internalization into macrophages are not clear.

A1: Thank you for your precious comments.

About the Ev characterizathion assays: Referring to previous papers have verified that *G. intestinalis* could secrete EVs, such as microvesicles, exosomes, large EVs and small EVs, we showed the GEVs morphology through transmission electron microscopy negative staining (figure 1A, line 305-308), measured the GEVs size through nanoparticle tracking analysis (figure 1B, line 308-310), explored the GEVs contents through proteomic assays (figure 1C and D, Table S1, S2 and S3, line 311-325). These three results were displayed in the main body of the revised manuscript. TEM negative staining results showed that GEVs displayed typical rounded or cup-shaped with membrane bilayer and the diameter was among 100~200 nm, which was similar to that reported by Moyano et al. 2019 (ref-26, figure 2E). Proteomic results showed that a total of 154 proteins were identified from the trophozoite stage with 49.4% (76/154) proteins containing at least two unique peptides (Table S1). Among them, 23 proteins were uncharacterized protein, and from the 131 remaining, 26 proteins and 46 proteins individually corresponded to previously identified in *G. duodenalis* microvesicles (ref-31) and large or small extracellular vesicles (ref-30). Proteins that involves in EVs biogenesis were existed in the GEVs, such as 14-3-3, alpha-tublin, heat shock proteins (ref-32). Proteins that involved in pathogenesis were overlaps with that identified in microvesicles, large and small extracellular vesicles in *G. duodenalis*, such as VSP, giardin, Arginine deiminase, Ornithine transcarbamylase, etc (ref-30, 31). Gene ontology (GO) annotations showed that 95 genes were related to immune response, 30 genes were associated with cell killing, 82 genes involved in transporter activity, etc. (Table S2, Figure 1C). The identified proteins may take part in 129 pathways, including Ribosome, Glycolysis, NOD-like receptor signaling pathway, MAPK signaling pathway, PI3K-Akt signaling pathway, Toll-like receptor signaling pathway, mTOR signaling pathway, etc. (Table S3, Figure 1D). Altogether, these data verified that we successfully isolated GEVs from *G. duodenalis* trophozoites.

Ref-26: Moyano S, Musso J, Feliziani C, Zamponi N, Frontera LS, Ropolo AS, et al. Exosome biogenesis in the protozoa parasite Giardia lamblia: a model of reduced interorganellar crosstalk. Cells. 2019;8(12): 1600.

Ref-30: Gavinho B, Sabatke B, Feijoli V, Rossi IV, Silva JM, Evans-Osses I, et al. Peptidylarginine deiminase inhibition abolishes the production of large extracellular vesicles from Giardia intestinalis, affecting host-pathogen interactions by hindering adhesion to host cells. Front Cell Infect Microbiol. 2020;10: 417.

Ref-31: Evans-Osses I, Mojoli A, Monguió-Tortajada M, Marcilla A, Aran V, Amorim M, et al. Microvesicles released from Giardia intestinalis disturb host-pathogen response in vitro. Eur J Cell Biol. 2017;96(2): 131-42.

Ref-32: Li S, Gong PT, Tai LX, Li X, Wang XC, Zhao CY, et al. Extracellular vesicles secreted by Neospora caninum are recognized by toll-Like teceptor 2 and modulate host cell innate immunity through the MAPK signaling pathway. Front immunol. 2018;9: 1633.

About the internalization assays: we read two papers on internalization experiments. Evans-Osses et al. reported that microvesicles released from *Giardia intestinalis* are captured by immature dendritic cells (iDCs) through assays of flow cytometry and confocal imaging. The plasma membrane vesicles (MVs) were labelled with PKH-67 and then added to iDCs. The percentage of PKH-67^+^ iDCs was analysed. This phenomenon was blocked by Cytochalasin-D. In addition, the MVs could be co-localized with proteins, such as early endocytosis associated protein. Ma’ayeh et al. reported that *Giardia intestinalis* excreted-secreted products (ESPs) were internalized to intestinal epithelial cells (IECs) by observing Alexa Fluor488-labelled ESPs location using confocal imaging assay. In our last manuscript version, we only pictured PKH-67 labeled GEVs in primary mouse peritoneal macrophages through confocal imaging assays at 1 h, 3 h, and 6 h. Considering those previous reports, we improved our *Giardia duodenalis* extracellular vesicles (GEVs) internalization assays as follows. First, we inoculated 25 μg of PKH67 labeled GEVs into macrophages and incubated for 1 h, 3 h, and 6 h and quantified the PKH67 positive cells percentage using flow cytometry. We found that PKH67-labeled GEVs could enter into macrophages in a time-dependent manner. Moreover, most GEVs were rapidly internalized into hosts within 3 h (79.2%). Then, we compared different doses of GEVs (25 μg or 12.5 μg) internalization into host cells and found that GEVs entered into macrophages in a dose-dependent manner. Finally, we determined the way of GEVs entrance into macrophages, cells were pretreated with Cytochalasin-D, then add labeled GEVs and observed with laser confocal. We found that GEVs may probably be captured by macrophages through active phagocytosis.

Reviewer 2

Q1: Line 287: Figure 1A and Figure 1b : EVs can not be clearly identified in the images. New images are required to confirm 1) EVs 2) Lipid Bilayer.

A1: Thank you for your precious comments. Both cup-shaped GEVs (may be exosomes) and rounded GEVs with membrane bilayer were existed in our negative staining results. After referring to Moyano et al. 2019, we replaced our negative staining results and double membrane were clear (figure 1A, line 305-308).

Q2: Lines 291 and 292: SEM images indicate that the trophozoites are not healthy or even viable, or may have been damaged in the SEM preparation process. New SEM images are required. Overall, SEM images don’t show evidence of EVs and TEM images might be showing debris.

A2: Thank you. We are wondering why the trophozoites are viable, in fact, they have been fully fixed in the coverslip. Structure of acetabulum and flagellum both existed. After repeating this assay (dislayed below), most of our results were consistent with previous results. Referring to “Extracellular vesicles in parasitic disease”, this paper showed SEM results of *Leishmania amazonensis* and *T. cruzi trypomastigotes*, and described that they are shedding EVs. Considering previous papers have verified that *G. intestinalis* could secrete EVs, such as microvesicles, exosomes, large EVs and small EVs, we delete SEM results to again verify that *G. intestinalis* could shed GEVs. Instead, to verify we successfully obtained GEVs, we showed the GEVs morphology through transmission electron microscopy negative staining (figure 1A, line 305-308), measured the GEVs size through nanoparticle tracking analysis (figure 1B, line 308-310), explored the GEVs contents through proteomic assays (figure 1C and D, Table S1, S2 and S3, line 311-325). These three results were displayed in the main body of the revised manuscript. Considering TEM results outside of trophozoites may be debris, we referred to Moyano et al. 2019 (ref-26) and found other TEM images were consistent withe that reported by Moyano et. The attached image illustrated that GEVs may also derive from intraluminal vesicles from endosome/lysosome peripheral vacuoles (ref-26). Considering the origin of GEVs has been verified, so we do not put this image in the main body of the manuscript.

TEM

Ref-26: Moyano S, Musso J, Feliziani C, Zamponi N, Frontera LS, Ropolo AS, et al. Exosome biogenesis in the protozoa parasite Giardia lamblia: a model of reduced interorganellar crosstalk. Cells. 2019;8(12): 1600.

SEM

Q3: Line 318: Do GEVs induce apoptosis or necropsy? Apoptosis could explain cytokine status, Macrophage engulfment of particles is common.

A3: Thank you. Previous studies by showed that Giardia can trigger enterocyte apoptosis, activated roapoptotic caspase-3 and -9, increased expression of proapop-totic Bax, decreased expression of antiapoptotic Bcl-2, and induced proteolytic cleavage of poly (-ADP-ribose) polymerase (PARP), finally induced alterations in the enterocyte tight junctions by breakdown/relocalization of proteins associated with these structures. Causing these genes or proteins have not been determined in our assays, so we do not explored the relation with apoptosis and necropsy. But NLRP3 inflammasome may probably trigger cell death of pyroptosis with active caspase 1 p20, moreover, KEGG analysis of GEVs showed that some proteins in GEVs may lead to necroptosis, thus, our group explored the expression of pyroptosis execution protein of GSDMD and found that GEVs indeed mediated pyroptosis in macrophages. This would be reported in detail after research in depth.

Ref: Certad G, Viscogliosi E, Chabe M, Caccio SM. Pathogenic mechanisms of *Cryptosporidium* and *Giardia*. Trends in Parasitology. 2017;33: 561-76.

Q4: Lines 296: General Comment: Figure legends should be at the end of the manuscripts (please amend in all the manuscript).

A4: Thank you. According to the guidelines for authors: “Insert figure captions in manuscript text, immediately following the paragraph where the figure is first cited (read order). Don’t include captions as part of the figure files themselves or submit them in a separate document”, we have to insert figure captions following the paragraph.

Q5: Line 334: Follow the nomenclature of the genes.

A5: Thank you. The gene names have been corrected following the nomenclature of the genes.

Q6: Lines 340-345 Please rephrase.

A6: Thank you. These sentences have been rephrased in line 372-381.

Q7: Line 344- “Obviously” should not be used.

A7: Thank you. Corrections have been made as suggested (line 380).

Q8: Line 361- Have the authors tried different concentrations?

A8: Thank you for your suggestion. We supplemented these assays and added different concentrations of EVs, including 25 μg/mL, 12.5 μg/mL in the revised figure 2, 3, 4, 5.

Q9: Line 363: Giardia might be causing apoptosis and necrosis of macrophages.

A9: Thank you. Previous studies by showed that Giardia can trigger enterocyte apoptosis, activated roapoptotic caspase-3 and -9, increased expression of proapop-totic Bax, decreased expression of antiapoptotic Bcl-2, and induced proteolytic cleavage of poly (-ADP-ribose) polymerase (PARP), finally induced alterations in the enterocyte tight junctions by breakdown/relocalization of proteins associated with these structures. Causing these genes or proteins have not been determined in our assays, so we do not explored the relation with apoptosis and necropsy. But NLRP3 inflammasome may probably trigger cell death of pyroptosis with active caspase 1 p20, moreover, KEGG analysis of GEVs showed that some proteins in GEVs may lead to necroptosis, thus, our group explored the expression of pyroptosis execution protein of GSDMD and found that GEVs indeed mediated pyroptosis in macrophages. This would been reported in detail after research in depth.

Ref: Certad G, Viscogliosi E, Chabe M, Caccio SM. Pathogenic mechanisms of *Cryptosporidium* and *Giardia*. Trends in Parasitology. 2017;33: 561-76.

Q10: Line 364: Change wording. (“Extremely obvious increased” does not make sense)

A10: Thank you. The “all extremely obvious increased” was replaced with “significantly increased” (line 406).

Q11: Line 366: Is TLR4 activated by Giardia?

A11: Thank you. Sorry for our unclear description. The mRNA level of TLR4 was both up-regulated in the GEVs-treated group (mRNA fold change value=1.28) and *G. duodenalis*-treated group (mRNA fold change value=1.84). This sentence has been rephrased as follows: “The mRNA level of TLR4 was significantly up-regulated only in the *G. duodenalis*-treated group (*p < 0.05), however, no significant change was observed in GEVs treated group.” line 410-411).

Q12: General comment = Please do not indicate mean and SD on the histograms. P vale could be added in the figure caption instead of the figure.

A12: Thank you. The mean, SD, and p value has been deleted as suggested.

Q13: Line 403-404: Please adjust the wording.

A13: Thank you. This sentence has been rephrased in line 500-502.

Q14: Line 405. The term “secretion” is not correct here. Also, there is no inhibition of cleavage of pro-IL-1 beta (Blockage of NLRP3 cleavage of pro IL-1 beta to IL-1b).

A14: Thank you. The secretion has been replaced with production (line 502). The WB results in the revised figure 6B were to detect the IL-1β p17 in the culturing supernatants (specified in line 511). Meanwhile, the pro-IL-1β in the cell lysate were not determined in this assay.

Q15: Line 421: What is the positive control? G. duodenalis? Please specify.

A15: Thank you. The positive control in the GEVs group was GEVs and in the *G. duodenalis* group was *G. duodenalis*. This has been specified in line 508-509.

Q16: Line 421: Figure 6 should be split into 2 figures as the message is confusing.

A16: Thank you. We split figure 6 into 2 figures, the mRNA levels of cytokines was re-displayed in figure S1. And cytokines secretion was re-displayed in the revised figure 6.

Q17: Line 426: caspase p20 should be caspase 1?

A17: Thank you. Corrections have been made as suggested (line 442).

Q18: Figure 7 could be presented before figure 6. Figure 7 displays NLRP3 inflammasome activation and figure 6 uses inhibitors (mechanism)

A18: Thank you. Corrections have been made as suggested.

Q19: Lines 445-446: Please rephrase.

A19: Thank you. This sentence has been rephrased as follows: “The pro-caspase1 protein level showed an up-regulated trend in a partially dose-dependent manner (**p < 0.01)” (line 461-462).

Q20: Line 782 (Figure 6 panel F). What is NC? Why NC is in GEV and G. duodenalis experiments? What is C? Positive control?

A20: Thank you. NC means negative control. In the untreated macrophages, no IL-1beta p17 was generated, which was used as a control for the inhibitor group. The C indicated positive control and replaced with PC. The NC and C have been specified in the legend of figure 6 (line 508-509).

Q21: Lune 784 (Figure 7) Panel A: Please specify that 6-, 12- and 24-hours treatments are for EVs. Also include size. Panel D: Specify that 12.5, 25 and 50ug are concentrations of EVs for clarity. Panel C&F : Specify band density in the legend. Also, please use cell lysate instead of LYS and SUP instead of SN.

A21: Thank you. GEVs were added in the revised figure 5A and D. The protein size was specified in the legends (line 473-474). The band density has been specified in the legend of figure 5C and F (line 477). The “cell lysate instead of LYS and SUP instead of SN” has been corrected in the revised figure 5A and D, and specified in the legends (line 474).

Q22: Line 788 (Figure 9). What is the line (the one before the conversion of IL-1beta to IL-1R) representing?

A22: Thank you. It the cell membrane.

Reviewer 3

Q1: Line 108. There are several publications dealing with Giardia EVs and also innate immune responses that are missing in the paper. They should be added both to the Introduction, Results and Discussion. See these papers:

Microvesicles released from Giardia intestinalis disturb host-pathogen response in vitro.

Evans-Osses I, Mojoli A, Monguió-Tortajada M, Marcilla A, Aran V, Amorim M, Inal J, Borràs FE, Ramirez MI. Eur J Cell Biol. 2017 Mar;96(2):131-142. doi: 10.1016/j.ejcb.2017.01.005. Epub 2017 Jan 22. PMID: 28236495

Characterization of the Giardia intestinalis secretome during interaction with human intestinal epithelial cells: The impact on host cells.

Ma'ayeh SY, Liu J, Peirasmaki D, Hörnaeus K, Bergström Lind S, Grabherr M, Bergquist J, Svärd SG. PLoS Negl Trop Dis. 2017 Dec 11;11(12):e0006120. doi: 10.1371/journal.pntd.0006120. eCollection 2017 Dec.

Peptidylarginine Deiminase Inhibition Abolishes the Production of Large Extracellular Vesicles From Giardia intestinalis, Affecting Host-Pathogen Interactions by Hindering Adhesion to Host Cells.

Gavinho B, Sabatke B, Feijoli V, Rossi IV, da Silva JM, Evans-Osses I, Palmisano G, Lange S, Ramirez MI.Front Cell Infect Microbiol. 2020 Sep 23;10:417. doi: 10.3389/fcimb.2020.00417. eCollection 2020.PMID: 33072615

Exosome Biogenesis in the Protozoa Parasite Giardia lamblia: A Model of Reduced Interorganellar Crosstalk.

Moyano S, Musso J, Feliziani C, Zamponi N, Frontera LS, Ropolo AS, Lanfredi-Rangel A, Lalle M, Touz M.Cells. 2019 Dec 9;8(12):1600. doi: 10.3390/cells8121600

A1: Thank you for your precious comments. These publications have been added in the introduction section (line 103-105, 111-115), results section (line 311-325, 334-336, 360-361), discussion section (line 561-568, 571-572, 578-580, 591-592) as suggested.

Q2：Line 286. The quality is poor in the EMs, compare eg with the images from the references above. I do not see a double membrane in the pictures provided and there are particles of different sizes.

A2: Thank you for your precious comments. Both cup-shaped GEVs (may be exosomes) and rounded GEVs with membrane bilayer were existed in our negative staining results. After referring to Moyano et al. 2019, we replaced our negative staining results and double membrane were clear (figure 1A, line 305-308). In the SEM results, there are different sizes of EVs. Referring to “Extracellular vesicles in parasitic disease”, this paper showed SEM results of *Leishmania amazonensis* and *T. cruzi trypomastigotes* with different sizes of EVs. To exclude the MVs’ interference, we filtered the collected culturing supernatants through a 0.22 µm sterilized PES membrane and the used for negative staining TEM observation and inoculation into macrophages for other assays. Considering that other papers have showed that *G. duodenalis* could secrete EVs and the origin of GEVs has been verified, and the aim of this study was to explore roles of GEVs, so we do not put TEM slide and SEM results in the main body of the manuscript.

Q3：Line 290. What is shown in the C panel?

A3: Thank you. The C panel indicates the concentration and diameter detection of GEVs using Nanosight. Reviewer 2 suggested that we should delete the C panel. Finally, we delete this panel.

Q4：Line 291. This is not convincing, one particle like structure from one cell, how do the authors know that the SEM is picking up EVs in this picture?

A4: Thank you. We referred to “Extracellular vesicles in parasitic disease”, this paper showed SEM results of *Leishmania amazonensis* and *T. cruzi trypomastigotes* shedding of EVs. So, we did this assays to observe whether *G. duodenalis* trophozoites could shed GEVs. Considering previous papers have verified that *G. intestinalis* could secrete EVs, such as microvesicles, exosomes, large EVs and small EVs, we delete SEM results to again verify that *G. intestinalis* could shed GEVs. Instead, to verify we successfully obtained GEVs, we showed the GEVs morphology through transmission electron microscopy negative staining (figure 1A, line 305-308), measured the GEVs size through nanoparticle tracking analysis (figure 1B, line 308-310), explored the GEVs contents through proteomic assays (figure 1C and D, Table S1, S2 and S3, line 311-325). These three results were displayed in the main body of the revised manuscript.

Q5: Line 294. Several papers have shown this already.

A5: Thank your for your advice. We have rephrased this sentence as follows: “Altogether, these data verified that we successfully isolated EVs from *G. duodenalis* trophozoites.” (line 324-325) and rephrased the title of this results as follows: “Characterization and proteomic analysis of GEVs” (line 304).

Q6: Line 309. I would be good to see just labelled EVs in super resolution.

A6: Thank you. We pictured with higher magnification on the “Effect of CytD on the internalization of GEVs into mouse peritoneal macrophages” using confocal microscopy. The labeled EVs are in super resulution (figure 2D). We hope this could make our results more clear.

Q7: Line 316. Can co-staining be done to see in what compartment the signal from PKH67 is seen?

A7: Thank you for your precious advice. From the provided references, we only found one paper that Evans-Osses et al. 2017 explored the co-staining PKH67 labeled MVs with Transferrin Receptor (TfR) or Early Endosome Antigen (EEA-1). This further verifying that the MVs were captured by iDCs through active phagocytosis. Considering this has been verified by Evans-Osses, in addition, researchers of Gavinho et al. 2020 or Moyano et al. 2019 have characterized that *G. duodenalis* could secrete extracellular vesicles through proteomic analysis or EM observation. We supplemented assays by blocking the phagocytosis with CytD and found that most GEVs could be blocked (figure 2). In addition, we supplemented the proteomic data (figure 1, Table S1, S2, S3). The aim of this study was to explore the role extracellular TLR2 recptor and intracellular NLRP3 inflammasome in regulating GEVs triggered inflammatory response, not the col-location of GEVs with which compartment in cells. We have to verify that the GEVs were obtained and GEVs could enter into macrophages. If it is still necessary to explore which compartment is co-localization with GEVs, we will buy these two antibodies mentioned by Evans-Osses to carry out the assay.

Q8: Line 319. How many macrophages were given 25ug EVs?

A8: Thank you. It is 5 × 10^5^ cells.

Q9: Line 326. Also secreted proteins (including EVs) induce chemokine expression in intestinal epithelial cells, see ref by Maayeh et al., 2017.

A9: Thank you. Corrections have been made as suggested (line 360).

Q10: Line 330. Is it certain that it cannot trigger any immune response?

A10: Thank you. Reviewer 2 told us that LAL assay measures LPS which is present in gram negative bacteria, not in the GEVs. Thus, it is not necessary to do this assay and we deleted this assay in the revised version.

Q11: Line 333. Why were not CCL20, CXCL 1-3 and CCL2 tested since they are induced in intestinal epithelial cells according to Ref 32? Addition of this would strengthen the paper and connect it to earlier data.

A11: Thank you. We read this paper “*Giardia lamblia*-induced changes in gene expression in diferentiated caco-2 human intestinal epithelial cells” and selected CCL20 and CXCL2 to detect its transcription levels after treatment with GEVs or inhibitors combined with GEVs. Results were displayed in the revised figure 3A and S1 and discussed in line 591-592, 617-620.

Q12：Line 334. Delete an extremely

A12: Thank you. Corrections have been made as suggested (ine 368).

Q13: Line 364. Delete all extremely obvious increased. Here it would be nice to test fixed trophozoites to see if they can induce a response.

A13: Thank you. The “all extremely obvious increased” was replaced with “significantly increased”. We explored the roles of GEVs and un-contacted *G. duodenalis* infection was used as control, which separate *G. duodenalis* from macrophages using an transwell, to exclude the influence of mechanical injury triggering by direct contact. Whether “fixed trophozoites” means un-contacted?

Q14: Line 374 and 377. Up-regulated does not mean that the receptors are activated.

A14: Thank you. Corrections have been made as suggested (line 402 and 417).

Q15: Line 388. It would be nice to see localization of TLR2 and NLRP3 in relationship to the ingested GEVs.

A15: Thank you. localization of TLR2 and NLRP3 in relationship to the ingested GEVs was supplemented in the revised figure 4C.

**Conclusions**

Reviewer 1

Q1: The experiments must be done with kinetics and dose dependence assays

A1: Thank you. Kinetics assays of GEVs internalization into macrophages and GEVs triggered NLRP3 inflammasome activation were displayed in the revised figure 2A and figure 5A-C. Dose dependence assays of GEVs internalization into macrophages, cytD inhibition GEVs internalization into macrophages, GEVs triggered inflammatory response, GEVs triggered TLRs and NLRs up-regulation, GEVs triggered NLRP3 inflammasome activation, GEVs triggered inflammatory response after inhibition of TLR2 and NLRP3 were displayed in the revised figure 2B, figure 2C-D, figure 3, figure 4A-B, figure 5 D-F, figure S1.

Reviewer 2

Q1: In the introduction section, the authors do not cite or discuss work done by other groups in the field of Giardia EVs. It would have been nice if they would have mentioned what other groups have shown regarding Giardia EVs and their involvement in various aspects of host parasite interactions including their effects on immune cells. For example: Evans-Osses et al. 2017 have shown that EVs can modulate dendritic cells

A1: Thank you. We have added research on *G. duodenalis* extracellular vesicles (EVs), including exosomes (line 103-105), large EVs and small EVs (line 111-113), microvesicles (line 113-115) in the introduction section and discuss their involvement in host parasite interactions.

Q2: Line 18 (Abstract): Giardia duodenalis is written in brackets as (G. duodenalis) which is incorrect.

A2: Thank you. The “(*G. duodenalis*)” has been replaced with “also known as *G. intestinalis* or *G. lamblia*” in line 18.

Q3: Line 39: Is the author summary required for Plos Neglected tropical diseases. If not, this section can be moved to the introduction.

A3: Thank you. Yes, the author summary section is required for Plos Neglected tropical diseases.

Q4: Line 55. Remove G. duodenalis in the brackets and instead of saying “also known”, please say “syn. G. lamblia, G. intestinalis”. Also, please note that only Assemblages A and B are zoonotic.

A4: Thank you. This sentence has been rephrased as suggested in line 55-56.

Q5: Line 60. Public health relevance: Giardia is not the main cause of Traveller’s diarrhea. Think of the many other common enteropathogens, such as EPEC.

A5: Thank you. This sentence “It is also known as traveler's diarrhea” has been deleted (line 60).

Q6: Line 61- References 6 and 7 are not the right references.

A6: Thank you. References 6 and 7 have been replaced with reference 4.

Q7: Line 66- A reference is needed for the statement that increasing resistance to drugs is becoming common.

A7: Thank you. References have been added in line 66 and 704-707.

Q8: Overall, the current article lacks some information which would have made it more compelling, and the conclusions better supported (eg. better imaging, proteomics, use of other inhibitors). The article does not include recent findings directly relevant to this study. Methods and results descriptions must be improved, as above.

A8: Thank you.

Better TEM imaging has been replaced in the revised figure1A.

Proteomics data have been added in figure1C and D, table S1, S2, S3.

The inhibitors mentioned above “Glyburide NLRP3 inhibitor is commonly used to inhibit NLRP3 inflammasome activation. Why did Glyburide not used in this study?”. Glibenclamide is also named Glyburide, they share the same CAS No. 10238-21-8. If it is necessary, we could replace the description of Glibenclamide with Glyburide.

Recent findings:

In the introduction section: we have added research on *G. duodenalis* extracellular vesicles (EVs), including exosomes (line 103-105), large EVs and small EVs (line 111-113), microvesicles (line 113-115) in the introduction section and discuss their involvement in host parasite interactions.

In the methods section: we referred to the *G. duodenalis* extracellular vesicles enrichment methods in line 142-143; carried out proteomic assays referring to ref-32 (line 163-173); labeled GEVs using PKH67 referring to ref-31 and 32 (line 183).

In the results section: compare GEVs proteomic data with that in *G. duodenalis* microvesicles and *G. duodenalis* large or small extracellular vesicles (line 313-316); compare GEVs internalization assays with *G. duodenalis* microvesicles internalization into dendritic cells and *G. duodenalis* large and small extracellular vesicles internalization into Caco-2 cells (line 334-336).

In the discussion section: discuss other reported *G. duodenalis* extracellular vesicles with GEVs and their functions/contents/capture ability (line 564-570, 573-582).

Reviewer 3

Q1: It is very important to use the correct references in order to make good conclusions.

A1: Thank you. Conclusions have been re-made in line 669-674.

Q2: Line 517. Reference earlier EV work in the discussion.

A2: Thank you. We discuss other reported *G. duodenalis* extracellular vesicles with GEVs and their functions/contents/capture ability (line 564-570, 573-582).

Q3: Line 529. Strange sentence.

A3: Thank you. This sentence has been deleted (line 592).

Q4: Line 558. Delete sentence, does not make sense.

A4: Thank you. This sentence has been deleted as suggested (line 624).

Q5: Line 564. The results are very different from the two studies. Why is this? Are there differences in parasite and mouse strains, GEVs vs trophozoites or what?

A5: Thank you. This might probably due to the different choices of inoculation ways. Li et al. used directly contacted models through adding trophozoites from *G. duodenalis* WB strain to macrophages. We used GEVs isolated from *G. duodenalis* WB strain to infect macrophages. Meanwhile, we set non-contacted models through adding trophozoites from *G. duodenalis* WB strain onto transwell and infect macrophages. This may lead to different functioning ways. Also, the infection doses may also lead to different results (line 626-628).

Q6: Line 568. See paper by Serradell showing that VSPs induce TLR2 and VSPs have been seen in EVs during proteomics (see earlier papers above).

A6: Thank you. This has been discussed in line 605-610 as follows: “ Serradell et al. reported that *Giardia* VSP1267 could active TLR4 and TLR2 receptors after replacement of he C-terminal transmembrane region and the cytoplasmic residues with a His purification tag 6. Moreover, our proteomic data of GEVs showed that several VSPs existed in the identified proteins and KEGG analysis showed that Toll-like receptor signaling pathway was activated”.

**Editorial and Data Presentation Modifications**

Reviewer #1: (No Response)

Reviewer #2:

Q1: I read with interest the research article by Zhao and colleagues dealing with the effects of Giardia EVs. The article is timely and investigates an important area of research in the field. The authors studied whether and how Giardia EVs may modulate the immune system. The investigators show that Giardia EVs (GEVs) may induce an immune/inflammatory response by increasing the production of cytokines. The effect was associated with the activation of TLR2 and NLRP3 inflammasome signaling pathways. The effects were diminished when macrophages were pre-treated with activation of TLR2 CA-074 methyl ester or zVAD-fmk. Overall, the current article lacks some information which would have made it more compelling.

A1: Thank you. The lacked information have been supplemented as suggested one by one.

Reviewer 3

Q1: The English has to be improved through-out the paper, there quality is relatively low now, starting already in the abstract.

A1: Thank you. We have checked the language throughout the manuscript and made some modifications in red.

Q2: Line 22. Explain EV and introduce GEV here.

A2: Thank you. Corrections have been made as suggested (line 20-21).

Q3: Line. Remove remarkably.

A3: Thank you. Corrections have been made as suggested (line 28).

Q4: Line 34. “Provide new targets against giardiasis” is not necessary.

A4: Thank you. This sentence has been deleted (line 34).

**Summary and General Comments**

Reviewer 1

Q1: the authors do not cite and do not know essential works in the area of Giardia and EVS. They do not consider manuscripts in giardiasis immunology. The authors must define experiments with two doses to know about dose dependence effect.

A1: Thank you for your precious comments. Several important literatures on *G. intestinalis* EVs or innate immune responses have been cited and in the introduction, results and discussion sections (highlighted in red). These publications are listed as follows:

Ref 26: Moyano S, Musso J, Feliziani C, Zamponi N, Frontera LS, Ropolo AS, et al. Exosome biogenesis in the protozoa parasite *Giardia lamblia*: a model of reduced interorganellar crosstalk. Cells. 2019;8(12): 1600.

Ref 30: Gavinho B, Sabatke B, Feijoli V, Rossi IV, Silva JM, Evans-Osses I, et al. Peptidylarginine deiminase inhibition abolishes the production of large extracellular vesicles from *Giardia intestinalis*, affecting host-pathogen interactions by hindering adhesion to host cells. Front Cell Infect Microbiol. 2020;10: 417.

Ref 31: Evans-Osses I, Mojoli A, Monguió-Tortajada M, Marcilla A, Aran V, Amorim M, et al. Microvesicles released from Giardia intestinalis disturb host-pathogen response *in vitro*. Eur J Cell Biol. 2017;96(2): 131-42.

Ref 36: Ma'ayeh SY, Liu J, Peirasmaki D, Hörnaeus K, Bergström Lind S, Grabherr M, et al. Characterization of the *Giardia intestinalis* secretome during interaction with human intestinal epithelial cells: the impact on host cells. PLoS Negl Trop Dis. 2017;11(12): e0006120.

Dose dependence assays of GEVs internalization into macrophages, cytD inhibition GEVs internalization into macrophages, GEVs triggered inflammatory response, GEVs triggered TLRs and NLRs up-regulation, GEVs triggered NLRP3 inflammasome activation, GEVs triggered inflammatory response after inhibition of TLR2 and NLRP3 were displayed in the revised figure 2B, figure 2C-D, figure 3, figure 4A-B, figure 5 D-F, figure S1.

Reviewer #2: as indicated above.

Reviewer #3:

Q1: Line 286. The quality is poor in the EMs, compare eg with the images from the references above. I do not see a double membrane in the pictures provided and there are particles of different sizes. Show enter examples.

A1: Thank you for your precious comments. Both cup-shaped GEVs (may be exosomes) and rounded GEVs with membrane bilayer were existed in our negative staining results. After referring to Moyano et al. 2019, we replaced our negative staining results and double membrane were clear (figure 1A, line 305-308). In addition, we referred to Moyano et al. 2019 and found other TEM images were consistent with that reported by Moyano et. The attached image illustrated that GEVs may also derive from intraluminal vesicles from endosome/lysosome peripheral vacuoles (ref-26). In the SEM results, there are different sizes of EVs. Referring to “Extracellular vesicles in parasitic disease”, this paper showed SEM results of *Leishmania amazonensis* and *T. cruzi trypomastigotes* with different sizes of EVs. To exclude the MVs’ interference, we filtered the collected culturing supernatants through a 0.22 µm sterilized PES membrane and the used for negative staining TEM observation and inoculation into macrophages for other assays. Considering the origin of GEVs has been verified, so we do not put this image in the main body of the manuscript.

TEM

Q2: Line 291. This is not convincing, one particle like structure from one cell, how do the authors know that the SEM is picking up EVs in this picture? Show more examples.

A2: Thank you. We referred to “Extracellular vesicles in parasitic disease”, this paper showed SEM results of *Leishmania amazonensis* and *T. cruzi trypomastigotes* shedding of EVs. So, we did this assays to observe whether *G. duodenalis* trophozoites could shed GEVs. Considering previous papers have verified that *G. intestinalis* could secrete EVs, such as microvesicles, exosomes, large EVs and small EVs, we delete SEM results to again verify that *G. intestinalis* could shed GEVs. Instead, to verify we successfully obtained GEVs, we showed the GEVs morphology through transmission electron microscopy negative staining (figure 1A, line 305-308), measured the GEVs size through nanoparticle tracking analysis (figure 1B, line 308-310), explored the GEVs contents through proteomic assays (figure 1C and D, Table S1, S2 and S3, line 311-325). These three results were displayed in the main body of the revised manuscript.

Q3: Line 309. I would be good to see just labelled EVs in super resolution microscopy.

A3: Thank you. We pictured with higher magnification on the “Effect of CytD on the internalization of GEVs into mouse peritoneal macrophages” using confocal microscopy. The labeled EVs are in super resulution (figure 2D). We hope this could make our results more clear.

Q4: Line 316. Can co-staining be done to see in what compartment the signal from PKH67 is seen?

A4: Thank you for your precious advice. From the provided references, we only found one paper that Evans-Osses et al. 2017 explored the co-staining PKH67 labeled MVs with Transferrin Receptor (TfR) or Early Endosome Antigen (EEA-1). This further verifying that the MVs were captured by iDCs through active phagocytosis. Considering this has been verified by Evans-Osses, in addition, researchers of Gavinho et al. 2020 or Moyano et al. 2019 have characterized that *G. duodenalis* could secrete extracellular vesicles through proteomic analysis or EM observation. We supplemented assays by blocking the phagocytosis with CytD and found that most GEVs could be blocked (figure 2). In addition, we supplemented the proteomic data (figure 1, Table S1, S2, S3). The aim of this study was to explore the role extracellular TLR2 recptor and intracellular NLRP3 inflammasome in regulating GEVs triggered inflammatory response, not the col-location of GEVs with which compartment in cells. We have to verify that the GEVs were obtained and GEVs could enter into macrophages. If it is still necessary to explore which compartment is co-localization with GEVs, we will buy these two antibodies mentioned by Evans-Osses to carry out the assay.

Q5: Line 333. Why were not CCL20, CXCL 1-3 and CCL2 tested since they are induced in intestinal epithelial cells according to Ref 32? Addition of this would strengthen the paper and connect it to earlier data.

A5: Thank you. We read this paper “*Giardia lamblia*-induced changes in gene expression in diferentiated caco-2 human intestinal epithelial cells” and selected CCL20 and CXCL2 to detect its transcription levels after treatment with GEVs or inhibitors combined with GEVs. Results were displayed in the revised figure 3A and S1 and discussed in line 591-593, 618-620.

Q6: Line 388. It would be nice to see localization of TLR2 and NLRP3 in relationship to the ingested GEVs.

A6: Thank you. localization of TLR2 and NLRP3 in relationship to the ingested GEVs was supplemented in the revised figure 4C.

Attached files (Giardia PNTD):

Q1: The manuscript Extracellular vesicles secreted by Giardia duodenalis regulate host cell innate immunity via TLR2 and NLRP3 inflammasome signaling pathways by Zhao et al. is an interesting work that must be improved to be accepted in PNTD.

The authors in their introduction and formulation of hypotheses suggest that there are no works describing extracellular vesicles and aspects of innate immunity in Giardia intestinalis. They did not consider the contribution of 3 groups from Swedish, Brazilian and Argentine citing exosomes and microvesicules of G intestinalis and its intearaction with the host. This information has been considered in at least 5 reviews in the parasite-host cell field and the first EV report in G intestinalis was four years ago!!!Even more the production and purification of EVs in the manuscript is unusual and nuclear.

A1: Thank you for your precious comments.

About the Giardia intestinalis extracellular vesicles background: In the introduction section, we have added research on *G. duodenalis* extracellular vesicles (EVs), including exosomes (line 103-105), large EVs and small EVs (line 111-113), microvesicles (line 113-115) in the introduction section and discuss their involvement in host parasite interactions.

About the production and purification of EVs: Up to now, several studies described the Giardia EVs isolation, including exosomes (by Moyano et al. 2019), microvesicles (by Evans-Osses et al. 2017) and large/small extracellular vesicles (by Gavinho et al. 2020). Among them, Evans-Osses and Gavinho are from the same team of Ramirez. Thus, they used similar methods to enrich EVs. In 2017, corresponding author of Ramirez reported that plasma membrane vesicles-microvesicles were produced from the *G. intestinalis* trophozoite. They enriched trophozoites to 1x10^6^ parasites/ ml in serum-free culture medium and stimulated with 1mM CaCl_2_ for 1h at 37ºC. The aim of this process was to stimulate MV production prevailed over exosome release in a short time. After low speed gradient centrifugation and ultracentrifugation, MVs were obtained. They obtained 11 kinds of proteins in the MVs. In 2020, corresponding author of Ramirez reported that extracellular vesicles (EVs) were produced from the *G. intestinalis* trophozoite. They used similar process to induce EVs production. After low speed gradient centrifugation, they first enriched LEV. After ultracentrifugation, they enriched SEV. There are 76 kinds of proteins in the LEV and 19 kinds of proteins in the SEV. There are 42 kinds of proteins both existed in the LEV and SEV. In 2019, another team by Moyano showed the enrichment method of exosomes. They let *G. intestinalis* trophozoite live in serum-free and bovine-free culture medium for 4 h, and then centrifugation with low speed, ultracentrifugation with high speed, isolation exosomes from EVs through sucrose density gradient centrifugation, and finally obtained purified exosomes. In our study, we want to enrich extracellular vesicles that secreted by the *G. intestinalis* trophozoite and do not exclude exosomes. Thus, we do not use stimulants of CaCl_2_. Combining the methods that enrich EVs from *Neospora caninum* (reported by Li et al.), we enriched EVs from the exosome-depleted fetal bovine serum TYI-S-33 medium cultured *G. duodenalis*. After low speed gradient centrifugation and ultracentrifugation, GEVs were obtained.

Ref:

Moyano S, Musso J, Feliziani C, Zamponi N, Frontera LS, Ropolo AS, et al. Exosome biogenesis in the protozoa parasite Giardia lamblia: a model of reduced interorganellar crosstalk. Cells. 2019;8(12): 1600.

Evans-Osses I, Mojoli A, Monguió-Tortajada M, Marcilla A, Aran V, Amorim M, et al. Microvesicles released from Giardia intestinalis disturb host-pathogen response in vitro. Eur J Cell Biol. 2017;96(2): 131-42.

Li S, Gong PT, Tai LX, Li X, Wang XC, Zhao CY, et al. Extracellular vesicles secreted by Neospora caninum are recognized by toll-Like teceptor 2 and modulate host cell innate immunity through the MAPK signaling pathway. Front immunol. 2018;9: 1633.

Q2: I also recomend that the group read a paper position of the international socitey of extracellular vesicles with the minimum requirements to characterize [EVs.The](https://ssl.microsofttranslator.com/bv.aspx?ref=TAns&from=&to=en&a=EVs.The) authors show Nano tracking data however they did not perform the reading of 3 measurements for one minute which is the normal procedure. The data presented is not convincing being difficult to accept.

A2: Thank you for your precious comments. Sorry for our unclear descriptions. We described the NTA protocol in detail. The NTA assay was carried out three times. Each time, the machine pictured 30 frames per second and lasted for 1 min.

Q3: Uptake trials in macrophages are not convincing too. In general Extracelular vesicles are made up of highly fuseable molecules and the internalizacao processes are very fast. Moreover Evans Osses et al 2017, Gavinho et al, 2020 have described uptake to eukariotic cell with reduzed amount of Gevs .25 ug de Evs is a huge concentration of EVs. Other authors have already shown na intense internalization with less GEVs

A3: Thank you for your precious comments. About the uptake trials in macrophages: referring to these published EVs internalization assays, we re-evaluated the capture ability of macrophages through labeling GEVs with PKH67 dye, which could specially combine with cell membrane, and then determined the amounts of PKH67 positive cells using flow cytometry . GEVs could be rapidly internalized into macrophages (19-33% PKH67+ cells) within 1 h, in contrast, G. intestinalis microvesicles were captured by immature dendritic cells (30-40% PKH67+ cells) within 30 minutes. Most GEVs could be internalized into macrophages within 3 h. The difference with that reported of microvesicles may attribute to the immune cell types, cell amounts or EVs amounts.

About the GEVs amount: referring to Evans Osses et al 2017 (figure 4B, 25 or 12.5 μg) and Li et al 2018 (figure 4, 50 μg/mL-25 μg per well), and considering the cell amounts, finally we use this this dose of GEVs.

Q4: In general the authors must justify because they used 25 ug of vesicles. In tempo 0 and at an hour of incubation there should be Evs at the plasmatic membrane. The controls at the internalization should not be PBS and should be endocitocis inhibitors like cythochalasin to demonstrate that the vesicles enter through passive or active processes. 25 ug is a lot of EVs and the data may be indicating that there are problems in the PKH labeled of EVs as already indicated by gavinho et al, 2020

A4: Thank you for your precious comments. We used 25 μg of GEVs by measuring with BCA protein assay kit. According to the suggestions by reviewer 1, time-dependent or dose-dependent internalization assays were quantified using flow cytometry and displayed in the revised figure 2A and B. Inhibition assays with cytochalasin D were carried out and displayed in the revised figure 2D. The labeled amount of EVs and procedure were referred to Li et al. 2018. About the GEVs amount: though gavinho et al used 7 or 14 μg of EVs, however, referring to Evans Osses et al 2017 (figure 4B, 25 or 12.5 μg) and Li et al 2018 (figure 4, 50 μg/mL-25 μg per well), and considering the cell amounts we used, finally we use this this dose of GEVs.

Li S, Gong PT, Tai LX, Li X, Wang XC, Zhao CY, et al. Extracellular vesicles secreted by Neospora caninum are recognized by toll-Like teceptor 2 and modulate host cell innate immunity through the MAPK signaling pathway. Front immunol. 2018;9: 1633.

Q5: The authors should justify because they work with 25 ml ug in all experiments and try to do experiments with different doses in order to show dose dependence effect .

A5: Thank you for your precious comments. Two or three doses of GEVs were carried out to explore the dose dependence effect in the revised figure 2, 3, 4, 5.

Q6: Again the authors did not cite the literature when they show pro-inflammatory effects of EVS release. It is known that giardiasis takes place with low cytokine release and not pro inflamatory release. There are still manuscript on the effect of the secretoma of G.I against eukaryotic cell.

A6: Thank you for your precious comments. In the introduction section, *G. duodenalis* trophozoites could influence the production of proinflammatory cytokines in WT mouse peritoneal macrophages (Li et al., line 76-78). In the discussion section, we discussed the chemokines mRNA levels changes with that reported by Ma'ayeh et al., 2017, which showed that *G. duodenalis* excretory-secretory products triggered intestinal epithelial cells chemokines transcription and release (line 589-592).

Ref:

Li X, Zhang XC, Gong PT, Xia FF, Li L, Yang ZT, et al. Tlr2−/− mice display decreased severity of giardiasis via enhanced proinflammatory cytokines production dependent on AKT signal pathway. Front. Immunol. 2017;8: 1186.

Ma'ayeh SY, Liu J, Peirasmaki D, Hörnaeus K, Bergström Lind S, Grabherr M, et al. Characterization of the Giardia intestinalis secretome during interaction with human intestinal epithelial cells: the impact on host cells. PLoS Negl Trop Dis. 2017;11(12): e0006120.

Q7: The effect of GEVS And inflamasome should be done with more controls and with diferent concentrations of GEVS

A7: Thank you for your precious comments. Roles of GEVs on the host inflammatory response were carried out with two or three dose of GEVs and each assay were set control in the revised figure 3, 4, 5.
